# High‐resolution quantitative trait loci mapping and pyramiding effects of candidate genes for plant height in soybean

**DOI:** 10.1002/tpg2.70207

**Published:** 2026-02-24

**Authors:** Dan Sha, Zhenzhen Zhang, Yongzhe Gu, Shengrui Zhang, Aimal Nawaz Khattak, Yitian Liu, Caiyou Ma, Meng Hu, Jimeng Niu, Linfeng Yu, Shibi Zhang, Azhar Iqbal, Ahsan Muhammad, Jing Li, Junming Sun, Rongxia Guan, Bin Li

**Affiliations:** ^1^ The State Key Laboratory of Crop Gene Resources and Breeding, The National Engineering Research Center for Crop Molecular Breeding, Institute of Crop Sciences Chinese Academy of Agricultural Sciences Beijing China; ^2^ The Key Laboratory of Soybean Biology (Beijing), Ministry of Agriculture and Rural Affairs, China‐Uruguay Joint Laboratory on Soybean Research and Innovation, Institute of Crop Sciences Chinese Academy of Agricultural Sciences Beijing China

## Abstract

Plant height is a crucial agronomic trait that significantly influences plant architecture and yield in soybean (*Glycine max* (L.) Merr.). Identifying major genes regulating plant height and developing closely linked molecular markers are crucial for breeding soybean cultivars with ideal architecture. In this study, a recombinant inbred line (RIL) population (F_2:7‐8_) developed from a cross between two soybean cultivars with contrasting plant heights was used to conduct quantitative trait loci (QTL) mapping for plant height across five environments based on a high‐density genetic linkage map. As a result, 13 QTL associated with plant height were identified on seven chromosomes. Among these, four QTL (*qPH‐5*, *qPH6‐1*, *qPH18*, and *qPH19‐2*) were consistently detected across multiple environments. Candidate genes for three stable QTL (*qPH6‐1*, *qPH18*, and *qPH19‐*2) with major effects on plant height were identified by annotating single‐nucleotide polymorphisms within the parental haplotypes, combined with analyses of gene expression patterns and biological functions. Consequently, *TCP13*, *Dt2*, and *Dt1* were predicted as strong candidate genes influencing plant height within these loci, respectively. Haplotype analyses within RIL population and across diverse soybean germplasm revealed that allelic variation in each of these genes significantly affected plant height. Moreover, different haplotype combinations of the three genes exhibited distinct phenotypic effects, indicating a pyramiding effect of these three genes on plant height. These findings will facilitate molecular breeding of soybean cultivars with ideal plant architecture.

## Core Ideas

- Thirteen quantitative trait loci (QTL) associated with soybean plant height were identified using 192 recombinant inbred lines.
- Four stable QTL for plant height were consistently detected across environments.
- *TCP13*, *Dt2*, and *Dt1* were predicted as strong candidate genes affecting plant height in soybean.
- Haplotype analyses revealed pyramiding effect of *TCP13*, *Dt2*, and *Dt1* on plant height.
- These findings facilitated developing cultivars with ideal plant architecture in soybean breeding programs.

## Plain Language Summary

Plant height is a key trait influencing plant architecture and crop yield. In this study, we utilized a recombinant inbred line (RIL) population derived from two soybean cultivars with contrasting heights to perform quantitative trait loci (QTL) mapping for plant height across five environments, employing a high‐density genetic linkage map. We identified four stable QTL (*qPH‐5*, *qPH6‐1*, *qPH18*, and *qPH19‐2*) over multiple environments. Within three of these QTL (*qPH6‐1*, *qPH18*, and *qPH19‐2*), which exhibited high phenotypic variance contributions, we predicted *TCP13*, *Dt2*, and *Dt1* as strong candidate genes. Haplotype analyses within the RIL population and across diverse soybean germplasm demonstrated that allelic variation in each gene significantly affected plant height. Furthermore, different combinations of haplotypes for these genes indicated a pyramiding effect on soybean plant height. These findings will facilitate the molecular breeding of soybean cultivars with ideal plant architecture.

**Abbreviations**
AFLP: amplified fragment length polymorphism
CV: coefficient of variation
ICIM: inclusive composite interval mapping
LOD: logarithm of odds
QTL: quantitative trait loci
RAD‐seq: restriction site‐associated DNA sequencing
RFLP: restriction fragment length polymorphism
RIL: recombinant inbred line
SNP: single‐nucleotide polymorphism
*TFL1*
: TERMINAL FLOWER 1
WGRS: whole‐genome resequencing

## 1 INTRODUCTION

Soybean (*Glycine max* (L.) Merr.) is one of staple crops that provides vegetable protein and oil for both humans and livestock (Duan et al., 2022). Moreover, soybeans contain bioactive compounds such as isoflavones, oligosaccharides, and saponins. These components help in mitigating health‐promoting effects through reducing risks of chronic diseases (e.g., cancer, osteoporosis, and cardiovascular disorders) while simultaneously exhibiting anti‐aging, anti‐obesity, and anti‐renal failure activities (Hsieh et al., 2020; I. S. Kim et al., 2021; Rowland et al., 2018). However, increasing global population and evolving dietary patterns have led to rising demand of soybean, requiring sustained yield improvements to mitigate supply‐demand imbalances (Ray et al., 2013). In this context, plant architecture has been recognized as a key decisive of crop yield per unit area. Proper plant architecture can improve fertilizer use efficiency and enhance light utilization, thereby ensuring yield stability under high‐density planting conditions. (Han et al., 2024; S. Lee et al., 2015; S. Liu et al., 2020). Historically, the first “Green Revolution” achieved reduced wheat plant height through the utilization of semi‐dwarfing alleles *Rht‐B1b* and *Rht‐D1b*, which inhibit gibberellin (GA) signaling pathways (Dong et al., 2023; Peng et al., 1999). Similarly, in rice, the *Semidwarf 1* (*SD1*) gene modulates GA biosynthesis to control plant height (Jiao et al., 2023). These genetic improvements enhanced crop lodging resistance and photosynthetic efficiency, leading to substantial yield increases (Hedden, 2003). Consequently, the First “Green Revolution” transformed global agricultural systems, leading to a marked rise in worldwide food production (Ameen & Raza, 2018). As a typical quantitative trait, plant height is controlled by multiple genes. Although environmental variables such as light, temperature, and soil pH influence soybean plant height, genetic factors play the dominant role in its regulation under given conditions (He et al., 2021; S. Lee et al., 2015; Yang et al., 2021). In soybean, plant height is mainly determined by two key components: internode number and internode length (S. Li et al., 2023). Reduced internode number correlates with lower planting density and impaired pod formation, whereas shortened internode length facilitates compact architecture, collectively leading to decreased plant height and modified yield potential (Pedersen & Lauer, 2004; Quijano & Morandi, 2023). Therefore, quantitative trait loci (QTL) mapping and candidate gene mining for soybean plant height are of great significance for research on ideal plant architecture and high‐yield breeding.

To date, 277 QTL associated with soybean plant height have been reported in the SoyBase database (https://www.soybase.org/). In earlier studies, traditional genetic markers (i.e., restriction fragment length polymorphism [RFLP], amplified fragment length polymorphism [AFLP], and simple sequence repeats [SSR]) were used for QTL mapping, and most of the loci were mapped on broad genomic regions with low‐throughput molecular markers. This limitation impeded precise determination of genomic coordinates and quantitative effects of QTL or genes regulating target traits (Singh et al., 2009; Yu et al., 2011). In contrast, single‐nucleotide polymorphisms (SNPs) represent the most abundant, stable, and simplest form of genomic variation (Stölting et al., 2013). Therefore, high‐throughput and high‐density SNP markers have been widely used in the construction of high‐density genetic maps and QTL mapping (Hu et al., 2021; Y. Tian et al., 2022; J. Wang et al., 2022). With the development of next‐generation sequencing technologies, molecular markers have transformed from traditional RFLP, random amplified polymorphic DNA, and AFLP to emerging breeding tools based on high‐throughput sequencing, such as SNP markers. These include whole‐genome resequencing (WGRS), restriction site‐associated DNA sequencing (RAD‐seq), specific‐locus amplified fragment sequencing (SLAF‐seq), single‐cell sequencing, and nanopore sequencing (Bawa et al., 2022; B. Li et al., 2014; X. Wang, et al., 2024; Y. Wang et al., 2021; Xu & Bai, 2015). Among these, WGRS‐based genotyping is considered the ultimate method for detecting all available genetic variation and polymorphisms in breeding populations (Bhat & Yu, 2021).

Recently, genes regulating soybean plant height, such as *PH13* (Qin et al., 2023), *Dt2* (Kou et al., 2021), and *LHY* (Cheng et al., 2019), were identified. Additionally, several pleiotropic genes that can simultaneously regulate both soybean plant height and flowering time have been discovered, including *Dt1*, *AP1*, *GmTOE4a*, and *GmGBP1* (L. Chen et al., 2020; J. Sun et al., 2023; Yue et al., 2021; Zhao et al., 2015). Despite extensive studies on loci associated with soybean plant height, few loci have been reproducibly identified across diverse environments or heterogeneous genetic backgrounds. Previously, we developed a recombinant inbred line (RIL) population from a cross between two soybean cultivars differing in plant height and constructed a high‐density genetic map based on WGRS (Agyenim‐Boateng et al., 2023; T. X. Liu et al., 2023). In present study, we performed QTL mapping for soybean plant height and identified strong candidate genes for plant height regulation. Haplotype analyses of these genes enable the pyramiding of superior alleles, offering potential for improving soybean plant height. These findings facilitate plant height‐related gene and marker discovery and provide theoretical insights for developing soybean varieties with ideal plant architecture.

**Core Ideas**

- Thirteen quantitative trait loci (QTL) associated with soybean plant height were identified using 192 recombinant inbred lines.
- Four stable QTL for plant height were consistently detected across environments.
- *TCP13*, *Dt2*, and *Dt1* were predicted as strong candidate genes affecting plant height in soybean.
- Haplotype analyses revealed pyramiding effect of *TCP13*, *Dt2*, and *Dt1* on plant height.
- These findings facilitated developing cultivars with ideal plant architecture in soybean breeding programs.

## 2 MATERIALS AND METHODS

### 2.1 Plant materials

The mapping population consisted of 192 F_7:8_ RILs derived from the cross between the cultivars Zhonghuang 35 (ZH35, maternal parent, tall) and Zhonghuang 13 (ZH13, paternal parents, and semi‐dwarf). Experiments were conducted at the Shunyi Experimental Station (N 40°13′, E 116°34′) in Beijing during 2020 and 2021; Beipuchang Experimental Station (N 40°13′, E 116°33′) in Beijing in 2022; and Changping Experimental Station (N 40°13′, E 116°12′) in Beijing in 2021 and 2023 (designated as 2020SY, 2021SY, 2022BPC, 2021CP, and 2023CP). Planting was conducted in 2.00 m long rows with 0.10 m and 0.50 m intra‐ and inter‐row spacing, respectively. Plants were sown in June and harvested in October. Planting and post‐planting operations were carried out following the recommended agronomic practices.

The plant height data used in this study were sourced from the publicly available SoyOmics database (https://ngdc.cncb.ac.cn/soyomics). Its Phenome module aggregates approximately 27,000 phenotypic records encompassing 115 traits from 2898 soybean accessions across multiple environments (Y. Liu et al., 2023). From this resource, we utilized plant height measurements for a total of 1484 accessions across six environments: two planting years (2013 and 2014) in Beijing and Shanxi, and 2 years (2014 and 2015) in Henan. Specifically, the composition of accessions planted at each location was highly consistent between years. Furthermore, a subset of 365 accessions was present in all six environments, forming a consistent genetic background for comparison.

All three field sites are located within the Huang‐Huai‐Hai ecoregion, sharing highly similar climatic and photoperiod conditions. Therefore, phenotypic differences across these site‐year combinations can be attributed primarily to genetic factors rather than environmental discrepancies. Plant height assessment and haplotype analysis were performed separately for each single environment.

### 2.2 Plant height measurement

The soybean plants grown across five environments were harvested at physiological maturity. For parental lines and each of 192 RILs, five individual plants were randomly selected for plant height measurement, and then the mean values were calculated. Plant height (cm) was defined as the vertical distance from the cotyledonary node to the apical meristem of the main stem (Yin et al., 2017).

### 2.3 QTL mapping for soybean plant height

Genotyping of the 192 RILs was performed based on WGRS, and a high‐density genetic map comprising 4879 bin markers has been constructed previously (Agyenim‐Boateng et al., 2023; T. X. Liu et al., 2023). With this genetic map, QTL mapping for plant height was conducted using the BIP module of IciMapping v4.1 software (http://www.isbreeding.net/) with the Inclusive Composite Interval Mapping (ICIM) method (Meng et al., 2015). This method defines QTL by a confidence interval flanked by two closely linked markers, designated as the left and right markers. The parameters included a genome‐wide scanning interval of 1 cM and a logarithm of odds (LOD) threshold determined by 1000 permutation tests at a 95% confidence level (Agyenim‐Boateng et al., 2023). The ICIM additive (ICIM‐ADD) method was applied to analyze phenotypic data from five environments. QTL naming were followed by the nomenclature “*q*” for quantitative locus, trait name (plant height), chromosome number, and the number of QTL detected on each chromosome for each trait (Mccouch et al., 1997). To identify stable QTL, we followed the criterion that QTL detected in at least two environments and located within a 5 cM interval on the same linkage group were considered as a single, stable locus. The genomic interval for each stable QTL was then defined as the region spanning from the leftmost to the rightmost marker across its detections.

### 2.4 Candidate gene mining for soybean plant height

The Wm82.a2.v1 genome of soybean was used as a reference, and then the genes were annotated within stable major effect QTL intervals through the Phytozome online platform (https://phytozome‐next.jgi.doe.gov/). The SnpEff variant annotation tool was used to predict the annotation and effects of the SNPs in the exon and promoter regions. Genes containing missense or upstream SNPs were selected as candidate genes. Next, expression profiles of these genes were then examined using RNA‐seq data from the ExpPattern module in SoyOmics (https://ngdc.cncb.ac.cn/soyomics/expression_tool/); those genes predominantly expressed in the shoot, particularly the shoot apex, were prioritized. These genes were further annotated against the nt, KOG, PFAM, and Panther databases to predict their functions and identify genes potentially involved in plant height regulation. The structure of the candidate gene was drawn using GSDS 2.0 (https://gsds.gao‐lab.org/), and corresponding nucleotide sequences were retrieved from the Phytozome online platform.

### 2.5 RNA extraction and RT‐qPCR assays

Total RNA was extracted from the shoot apex of two parental cultivars (ZH35 and ZH13) using the RNA Easy Fast Plant Tissue Kit (DP452; Tiangen Biotech). Full‐length cDNA was synthesized from the extracted RNA with a cDNA synthesis kit (AE311; TransGen Biotech) and subsequently used as template for RT‐qPCR following the manufacturer's protocol (AQ101; TransGen Biotech). The soybean gene *GmActin6* (GenBank accession: NM_001289231) served as an internal control. Expression levels of *Dt1*, *Dt2*, and *TCP13* were quantified using the 2^−ΔΔCt^ method relative to the internal control. All primers used are listed in Table S1. The RT‐qPCR assays included three biological replicates, each with three technical replicates.

### 2.6 Haplotype analysis of candidate genes

Based on genotype data of key SNPs in candidate genes retrieved from SoyOmics (https://ngdc.cncb.ac.cn/soyomics/haplotype/), combined with plant height and pod number phenotypic data from both the RIL population and soybean accessions, we detected haplotype effects of three candidate genes and analyzed their pyramiding effects. Haplotype effect analysis was performed using analysis of variance (ANOVA) in IBM SPSS Statistics 26.0 software, with a significance threshold set at *p* < 0.05. Fisher's least significant difference multiple comparison post hoc test was employed. Results were visualized through GraphPad Prism 9.0 software (https://www.graphpad.com/).

### 2.7 Statistical analysis

The coefficient of variation (CV), environmental variance, genotypic variance, and broad‐sense heritability (*h* ^2^) were calculated using Microsoft Excel 2016. Kurtosis and skewness were calculated using the IBM SPSS Statistics 26.0 software (https://www.ibm.com/cn‐zh/spss). Changes in soybean plant height among parents and RIL populations across five environments were analyzed using Origin software (https://www.originlab.com/).

## 3 RESULTS

### 3.1 Phenotypic and genetic variation analysis of the RIL population

**FIGURE 1:**
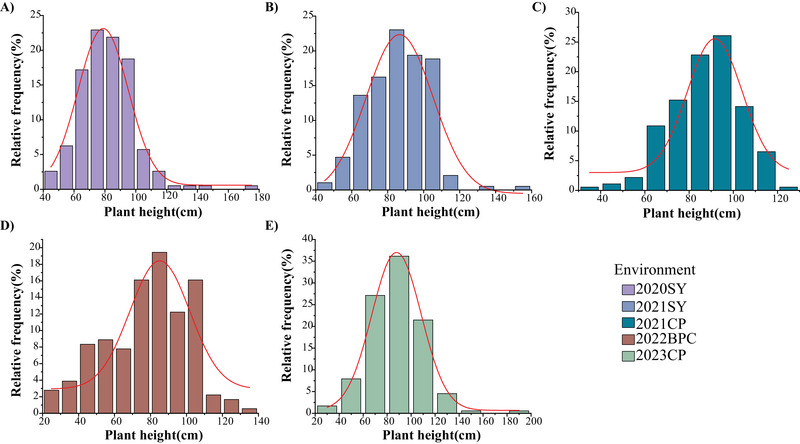
Frequency distribution of plant height across five environments in the recombinant inbred line (RIL) population. (A) 2020SY; (B) 2021SY; (C) 2021CP; (D) 2022BPC; (E) 2023CP.

Phenotypic evaluation of soybean plant height was conducted for ZH35 and ZH13, as well as their derived RIL population across five environments (Table 1). For the parental lines, ZH35 exhibited significantly greater plant height than ZH13 over four of the environments (2020SY, 2021SY, 2021CP, and 2022BPC), with average plant height of 87.20 and 69.23 cm, respectively. The RIL population exhibited extensive genetic variation, with plant height ranging from 22.25 to 192.80 cm and a mean of 84.15 cm. The CV ranged from 17.42% (2021SY) to 29.66% (2022CP) across environments (Table 1). Transgressive segregation was consistently observed across all environments, indicating contributions of additive alleles from both parents. ANOVA revealed significant effects of genotype, environment, and genotype‐by‐environment interaction on soybean plant height. The *h* ^2^ of soybean plant height across five environments was estimated at 95.86%, indicating that genetic factor predominantly governs this trait (Table 1). Frequency distribution analysis revealed a continuous variation in plant height among RILs. Shapiro–Wilk normality tests indicated that plant height followed normal distribution over three environments (2020SY, 2021CP, and 2023CP), while deviations from normality were observed in 2021SY and 2022BPC. These results align with the polygenic nature of plant height, a classic quantitative trait controlled by multiple loci (Figure 1).

**TABLE 1:** Phenotypic statistics of soybean plant height across five environments.

Traits	Environment	Parent		Minimum	Maximum	Mean	SD	CV(%)	*p* values from ANOVA			*h* ^2^ (%)	Shapiro–Wilk		
		ZH35	ZH13						G	E	G × E		Kurtosis	Skewness	*p‐*value
PH	2020SY	83.00	64.40	43.00	174.20	81.53	17.83	21.87	0.00E+00***	4.09E‐188***	0.00E+00***	95.86	3.50	0.98	0.282
PH	2021SY	94.42	68.73	43.28	151.01	85.63	16.50	19.27					0.58	0.12	0.018
PH	2021CP	99.90	85.26	39.15	122.30	87.50	15.25	17.42					−0.06	−0.3	0.369
PH	2022BPC	73.90	51.00	22.25	135.46	78.76	23.36	29.66					−0.37	−0.36	0.026
PH	2023CP	84.80	76.80	29.75	192.80	87.31	23.11	26.46					1.84	0.52	0.426

Abbreviations: ANOVA, analysis of variance; CV, coefficient of variation; E, environment; G, genotype; *h* ^2^, heritability; PH, plant height; SD, standard deviation.

***Significance at *p* < 0.001 level.

### 3.2 QTL mapping for soybean plant height

**FIGURE 2:**
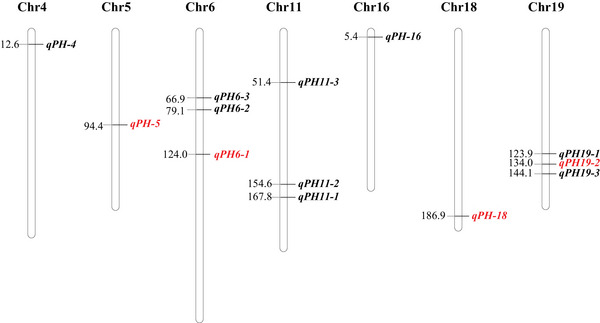
Genetic position of quantitative trait loci (QTL) for soybean plant height in the recombinant inbred line (RIL) population across five environments. Stable QTL detected across multiple environments were depicted in red color.

QTL mapping for soybean plant height was performed using the ICIM‐ADD method with an LOD threshold of 3.64. A total of 13 QTL were detected on chromosomes 4, 5, 6, 11, 16, 18, and 19, respectively (Figure 2). The LOD values of these QTL ranged from 2.62 to 62.68, with the individual QTL explaining 0.96%–33.88% of phenotypic variance (Table S2). QTL located on the same chromosome that were overlapping or in close position (<5 cM) across different environments were merged, resulting in four stable QTL, *qPH5*, *qPH6‐1*, *qPH18*, and *qPH19‐2* (Table 2). The additive effects of *qPH5*, *qPH6‐1*, and *qPH18‐1* were negative, indicating that the beneficial alleles originated from paternal parent ZH13, whereas positive additive effect of *qPH19‐2* suggests that favorable allele was originated from maternal parent ZH35. Therefore, the favorable alleles for plant height were contributed by both parents. *qPH5* was consistently detected in 2021SY and 2022BPC, explaining 0.97% and 2.73% of the phenotypic variance, respectively. This locus spans a genetic interval of 1.09 cM, with a physical distance of 164.35 kb. *qPH6‐1* was identified across three environments (2020SY, 2021SY, and 2022BPC), exhibiting a genetic distance of 2.45 cM with a physical span of 798.39 kb; it accounted for an average of 15.93% of phenotypic variance. *qPH18* showed consistent detection in 2020SY, 2022BPC, and 2023CP, covering a genetic interval of 3.49 cM with a physical distance of 808.12 kb. This locus explained an average of 4.72% of phenotypic variance for plant height. *qPH19‐2* demonstrated stability across all five environments, spanning 4.98 cM (with a physical distance of 593.04 kb) and explaining an average of 21.70% of the phenotypic variance. Among these four stable QTL, *qPH6‐1*, *qPH18*, and *qPH19‐2* exhibited relatively higher phenotypic variance explained values, suggesting their potential to harbor major‐effect genes regulating plant height in soybean. Therefore, we focused candidate gene mining specifically within these three QTL intervals.

**TABLE 2:** Stable quantitative trait loci (QTL) for soybean plant height across five environments.

QTL name	Environment	Chr.	Left marker	Right marker	LOD	PVE (%)	Add	Position (cM)
*qPH5*	2021SY	5	bin1160	bin1159	3.14	2.73	−3.07	94
	2022BPC	5	bin1160	bin1159	3.09	0.97	−3.91	94
*qPH6‐1*	2021SY	6	bin1429	bin1428	10.2	9.69	−5.76	126
	2020SY	6	bin1430	bin1429	10.91	5.22	−5.78	125
	2022BPC	6	bin1431	bin1430	62.68	32.87	−26.65	124
*qPH18*	2023CP	18	bin4544	bin4545	5.47	8.19	−6.18	187
	2022BPC	18	bin4548	bin4549	5.37	1.68	−5.13	188
	2020SY	18	bin4551	bin4552	9.11	4.29	−5.23	190
*qPH19‐2*	2021SY	19	bin4811	bin4812	28.35	33.88	10.84	136
	2021CP	19	bin4811	bin4812	7.28	10.16	6.11	136
	2023CP	19	bin4813	bin4814	14.89	26.02	11.1	137
	2022BPC	19	bin4813	bin4814	34.43	16.53	16.23	137
	2020SY	19	bin4814	bin4815	34.39	21.89	12.02	138

*Note*: QTL was designated as “*qPH*” (PH‐ representing plant height, followed by the chromosome number and their order).

Abbreviations: Add, additive effect; Chr., chromosome; cM, centimorgan; LOD, logarithm of odds; PVE, phenotypic variance explained.

### 3.3 Candidate gene mining within *qPH6‐1*, *qPH18*, and *qPH19‐2* genomic intervals

The candidate genes for the three major stable QTL were selected through a multi‐step approach. First, we focused on genetic variants likely to impact gene function, specifically upstream and missense SNPs. Next, we analyzed the expression profiles of these genes using RNA‐seq data from the SoyOmics database, prioritizing those predominantly expressed in the shoot, particularly the shoot apex. Finally, we considered functional annotations to identify genes potentially involved in plant height regulation.

Within the genomic region of *qPH6‐1*, a total of 28 genes were annotated (Table S3). Based on parental resequencing analysis, three of the 28 genes harbored upstream or missense SNPs (Table S4). Similarly, the genomic region of *qPH18* contained 139 annotated genes (Table S3), in which 107 genes harbored nonsynonymous, stop‐gained, and upstream SNPs (Table S4). Additionally, the genomic region of *qPH19‐2* contained 83 annotated genes (Table S3), with 24 genes harboring nonsynonymous and upstream SNPs (Table S4).

The expression patterns of these genes harboring upstream and missense SNPs were then examined using the RNA‐seq data in SoyOmics to select genes with predominant shoot expression. This step refined the candidate gene list to 2, 65, and 12 genes for *qPH6‐1*, *qPH18*, and *qPH19‐2*, respectively (Table S5).

**FIGURE 3:**
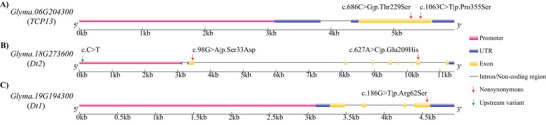
Gene structure of the three strong candidate genes for plant height, showing promoter, untranslated region (UTR), exon, intron, and vital single‐nucleotide polymorphism (SNP) variation positions between two parental lines of the recombinant inbred line (RIL) population. The arrows indicate the variation positions, and the SNP and amino acid variations between two parents are shown above the arrows.

In combination with gene function annotation, we further predicted strong candidate genes for these three loci. For *qPH6‐1*, functional annotation revealed that *Glyma.06G204300*, encoding the transcription factor *TCP13*, is particularly noteworthy. The *TCP* gene family serves as key regulators of cell proliferation and plays crucial roles in plant developmental processes, including leaf and floral organ morphogenesis, hypocotyl elongation modulation, and plant branching determination (Feng et al., 2018; Z. Wang et al., 2024; Xia et al., 2022). Therefore, *Glyma.06G204300* was predicted as the strong candidate gene controlling soybean plant height in this locus. Notably, two distinct amino acid substitutions were identified between ZH13 and ZH35: a C/G variation at genomic position Gm06:19212609 causes a Thr/Ser substitution, while a C/T variation at Gm06:19212986 results in a Pro to Ser alteration (Table 3; Figure 3).

**TABLE 3:** Functional single‐nucleotide polymorphism (SNP) variations in three strong soybean plant height candidate genes among parental lines.

Gene locus	Functional annotation	Position	ZH35	ZH13	Variation	Position of amino acids	Amino acid transition
*Glyma.06G204300*	*TCP13*	19212609	C	G	Nonsynonymous	229	Thr/Ser
		19212986	C	T	Nonsynonymous	355	Pro/Ser
*Glyma.18G273600*	*Dt2*	55638538	G	A	Nonsynonymous	33	Ser/Asp
		55645486	A	C	Nonsynonymous	209	Glu/His
		55635192	C	T	Upstream variant	–	–
*Glyma.19G194300*	*Dt1*	45184804	C	A	Nonsynonymous	62	Arg/Ser

Similarly, functional annotation of candidate genes within the *qPH18* interval identified *Dt2* (*Glyma.18G273600*), a MADS‐box transcription factor regulating soybean stem growth habit. This gene modulates plant height through stem growth regulation and plays essential roles throughout the plant lifecycle (Ping et al., 2014; D. Zhang et al., 2019). Therefore, *Dt2* was predicted as the candidate gene in this locus. Three functional SNPs were detected in *Dt2*: a G/A variation (Gm18:55638538) causing a Ser to Asp substitution, an A/C variation (Gm18:55645486) causing a Glu to His substitution, and a C/T polymorphism in promotor region (Gm18:55635192) (Table 3; Figure 3). According to these variations, ZH13 harbored *dt2* allele, while ZH35 harbored *Dt2* allele. The locus *qPH19‐2* was consistently detected across all environments, explaining up to 21.70% of the phenotypic variance in plant height on average, suggesting that this interval likely contains a major‐effect gene. Among the 12 genes characterized by harboring both upstream and missense variations between parental lines and by predominant shoot expression, we identified *Dt1* (*Glyma.19G194300*), a key regulator of soybean stem growth habit. In Arabidopsis, the homolog of *Dt1* encodes the flowering repressor TERMINAL FLOWER 1 (*TFL1*), which inhibits flowering and maintains indeterminate growth of the shoot apical meristem (X. Li et al., 2024; B. Liu et al., 2010; Z. Tian et al., 2010). In soybean, *Dt1* is a *TFL1* homolog and a well‐characterized regulator of plant height, pod‐setting habit, and flowering time, ultimately influencing yield potential (Miranda et al., 2020; Yue et al., 2021). Therefore, *Dt1* is proposed as key candidate gene underlying *qPH19‐2*. A functional SNP was identified in the exon of *Dt1*: a C/A variation (Gm19:45184804) causing an Arg to Ser substitution between ZH35 and ZH13 (Table 3; Figure 3), which leads to a *Dt1/dt1* difference.

Therefore, *TCP13*, *Dt2*, and *Dt1* emerged as strong candidate genes due to their established functional links to plant height, whereas the other genes on the list showed no clear or only tenuous connections.

The expression levels of these three strong candidate genes in the shoot apex of the two parental lines (ZH35 and ZH13) were further analyzed. As shown in Figure S1 the expression levels of *Dt2* and *TCP13* differed significantly between the parents. In contrast, *Dt1* expression was very low in both parents and showed no significant difference (Figure S1).

By integrating this multi‐faceted evidence, we propose *Dt1*, *Dt2*, and *TCP13* as strong candidate genes underlying *qPH19‐2*, *qPH18*, and *qPH6‐*1, respectively, for plant height regulation in soybean.

### 3.4 Haplotype analyses of the three candidate genes in the RIL population and soybean accessions

We then performed haplotype analyses across two populations, the 192 RILs and 1484 soybean accessions, to investigate associations between variations in *Dt1*, *Dt2*, *TCP13*, and soybean plant height.

**FIGURE 4:**
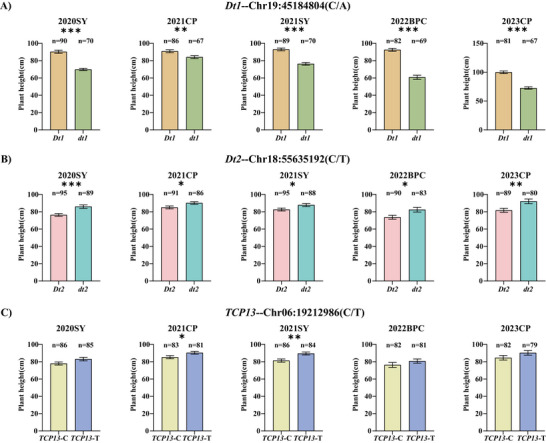
Haplotype analyses of three strong candidate genes for soybean plant height in the recombinant inbred line (RIL) population across five environments. (A) *Dt1*, (B) *Dt2*, and (C) *TCP13*. The bars show the plant heights of major haplotypes (more than five accessions) with standard errors (SE). The five environments were shown as 2020SY, 2021CP, 2021SY, 2022BPC, and 2023CP. *, **, *** indicate significance at *p* < 0.05, *p *< 0.01, and *p *< 0.001 levels.

In the RIL population, the *Dt1* coding region exhibited two haplotypes differentiated by the SNP at Gm19:45184804: the reference haplotype *Dt1* (CC) and alternative haplotype *dt1* (AA). Multi‐environment phenotypic analysis revealed consistent plant height differences between haplotypes. Across five environments, *Dt1* plants showed significant height advantages over *dt1* (7.46%–34.28% increase, with an average of 21.93%) (Figure 4A). For *Dt2*, we focused on the functionally characterized SNP in promoter, Gm18:55635192, a known regulatory site affecting gene function (Liang et al., 2022). Parental SNP divergence defined two haplotypes: *Dt2* (C) and *dt2* (T). The *dt2* accessions displayed reversed phenotypic trends, exhibiting 5.69%–11.16% greater plant height than *Dt2* across environments, with an average of 8.93% (Figure 4B). The *TCP13* coding SNP (Gm06:19212986), which has pronounced effect on its function (Xia et al., 2022), segregated into *TCP13*‐C and *TCP13*‐T haplotypes. *TCP13*‐T demonstrated superior plant height in two environments (2021CP: 5.17%, 2021SY: 8.12%), with an average increase of 6.23% over *TCP13*‐C (Figure 4C).

**FIGURE 5:**
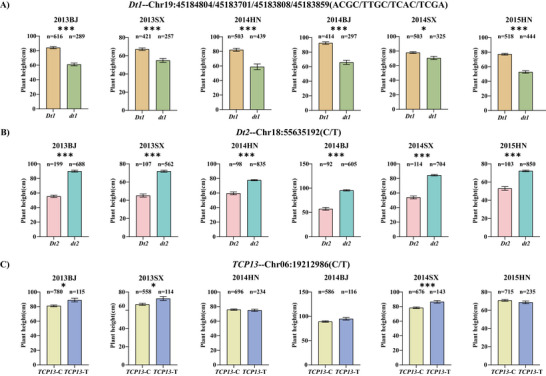
Haplotype analyses of three strong candidate genes for soybean plant height in the 1484 soybean accessions across six environments. (A) *Dt1*, (B) *Dt2*, and (C) *TCP13*. The bars show the plant heights of major haplotypes (more than five accessions) with standard errors (SE). The six environments were shown as 2013BJ, 2013SX, 2014HN, 2014BJ, 2014SX, and 2015HN. * indicates significance at *p* < 0.05 level, *** indicates significance at *p *< 0.001 level.

In the 1484 soybean accessions, the *Dt1* gene harbors four nonsynonymous SNPs. Compared with the Williams 82 reference genome, variations of varying degrees occur at sites Gm19:45184804, Gm19:45183701, Gm19:45183808, and Gm19:45183859. Moreover, a mutation at any one of these four sites can convert the semi‐dominant allele *Dt1* into the recessive allele *dt1* (Kou et al., 2021). Therefore, the *Dt1* gene has two types of haplotypes at these four sites: (i) the reference haplotype *Dt1* (TCGC), which is identical to the Williams 82 reference genome at all four sites, and (ii) the variant haplotypes *dt1* (ACGC, TTGC, TCAC, TCGA), each of which contains at least one non‐reference allele. Combined with multi‐year and multi‐site phenotypic data on plant height in soybean germplasm resources, the haplotype *Dt1* was significantly taller than the haplotype *dt1* in six independent environments, with increases of 27.39%, 28.66%, 18.29%, 9.47%, 28.40%, and 31.51%, respectively. The average plant height difference between the two haplotypes was 23.96% (Figure 5A). For the *Dt2*, two haplotypes at Gm18:55635192 also exhibited significant difference in plant height across soybean accessions. In six environments, the plant height of the *dt2* haplotype was significantly higher than that of the *Dt2* haplotype, with increases of 38.39%, 39.91%, 37.08%, 36.08%, 23.26%, and 26.67%, respectively. The average difference in plant height between the two haplotypes was 33.57% (Figure 5B). Regarding the *TCP13* gene, two haplotypes at Gm06:19212986 also exhibited significant difference among the soybean accessions. The plant height of the *TCP13*‐T haplotype was significantly higher than that of the *TCP13*‐C haplotype in three environments (2013BJ, 2013SX, and 2014SX), explaining 8.92%, 8.76%, and 9.41% of the phenotypic variation in plant height, respectively. The average difference in plant height between the two haplotypes was 9.03% (Figure 5C).

### 3.5 Pyramiding effect of the three candidate genes on plant height

To elucidate the breeding potential of these alleles, joint haplotype analysis was conducted between *Dt1* and *Dt2* and among *Dt1*, *Dt2*, and *TCP13* in the RIL population and 1484 soybean accessions.

**FIGURE 6:**
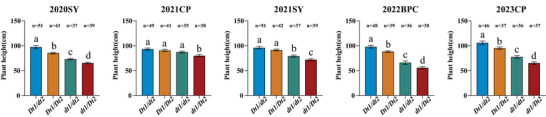
Pyramiding effect of *Dt1* and *Dt2* on soybean plant height in the recombinant inbred line (RIL) population. The bars show the plant heights of major haplotypes (more than five accessions) with standard errors (SE). Different lowercase letters above bars denote statistically significant differences at *p* < 0.05.

In the RIL population, joint haplotype analysis was conducted between *Dt1* and *Dt2*. Based on the two essential SNPs of these two genes, four possible combinations of growth habit were detected: the indeterminate growth habit combination *Dt1/dt2* (CT), the semi‐determinate combination *Dt1/Dt2* (CC), and the determinate growth combinations *dt1/Dt2* (AC) and *dt1/dt2* (AT). The combined haplotype effects of these combinations significantly differed across multiple environments. The indeterminate growth habit combination *Dt1/dt2* exhibited the highest plant height, followed by the semi‐determinate growth habit combination *Dt1/Dt2* and the determinate growth habit combination *dt1/d2* and *dt1/D2*. Interestingly, for two determinate growth habit combination, the plant heights of *dt1/Dt2* were significantly lower than that of *dt1/dt2* over five environments (Figure 6).

**FIGURE 7:**
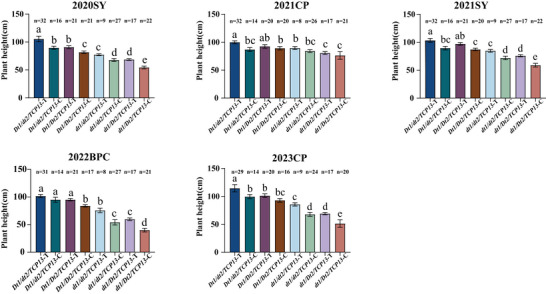
Pyramiding effect of *Dt1*, *Dt2*, and *TCP13* on soybean plant height in the recombinant inbred line (RIL) population. The bars show the plant heights of major haplotypes (more than five accessions) with standard errors (SE). Different lowercase letters above bars denote statistically significant differences at *p* < 0.05.

Based on the three SNPs in *Dt1*, *Dt2*, and *TCP13*, eight haplotype combinations were detected among the 192 RILs: *Dt1/Dt2/TCP13*‐C (CCC), *Dt1/Dt2/TCP13*‐T (CCT), *Dt1/dt2/TCP13*‐C (CTC), *Dt1/dt2/TCP13*‐T (CTT), *dt1/Dt2/TCP13*‐C (ACC), *dt1/Dt2/TCP13*‐T (ACT), *dt1/dt2/TCP13*‐C (ATC), and *dt1/dt2/TCP13*‐T (ATT). We observed that *TCP13* significantly affects plant height except for *Dt1* and *Dt2*, with the plant height of haplotype combinations containing *TCP13*‐T being significantly higher than those containing *TCP13*‐C in indeterminate, semi‐determinate, and determinate growth habit groups (Figure 7).

**FIGURE 8:**
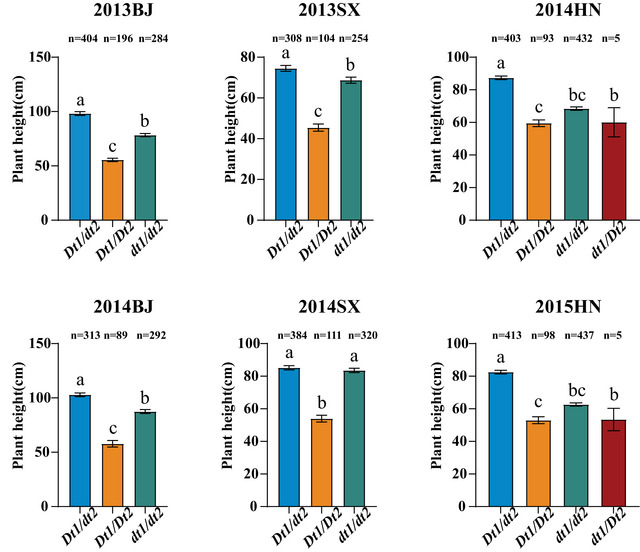
Pyramiding effect of *Dt1* and *Dt2* on soybean plant height in the 1484 soybean accessions. The bars show the plant heights of major haplotypes (more than five accessions) with standard errors (SE). Different lowercase letters above bars denote statistically significant differences at *p* < 0.05.

Joint haplotype analyses of *Dt1*, *Dt2*, and *TCP13* were also conducted in 1484 soybean accessions across six environments. First, pairwise analysis of *Dt1* and *Dt2* revealed four haplotype combinations: indeterminate (*Dt1/dt2*), sub‐determinate (*Dt1/Dt2*), and determinate types (*dt1/Dt2*, *dt1/dt2*). Of these haplotype combinations, *dt1/Dt2* is rare allele compared with other alleles, with allele frequencies ranging from 0.34% to 0.54% across six environments. The *Dt1/dt2* haplotype (indeterminate growth habit) exhibited taller plants than *Dt1/Dt2* (semi‐determinate growth habit) across six environments. For determinate growth habit haplotypes, *dt1*/*dt2* accessions were slightly higher than *dt1*/*Dt2* over 2014HN and 2015HN, although the difference is not significant (Figure 8).

**FIGURE 9:**
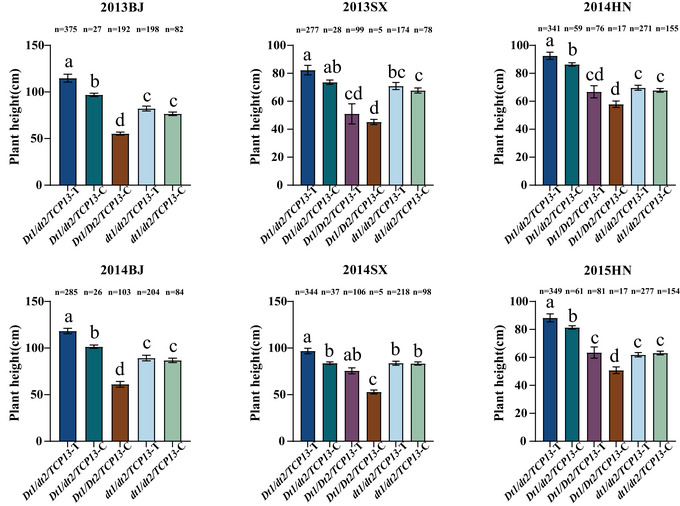
Pyramiding effect of *Dt1*, *Dt2*, and *TCP13* on soybean plant height in the 1484 soybean accessions. The bars show the plant heights of major haplotypes (more than five accessions) with standard errors (SE). Different lowercase letters above bars denote statistically significant differences at *p *< 0.05.

Referring to joint haplotype analysis among *Dt1*, *Dt2*, and *TCP13* in 1484 soybean accessions, eight combinations were detected: the indeterminate growth habit combinations *Dt1/dt2/TCP13*‐C and *Dt1/dt2/TCP13*‐T, the semi‐determinate growth habit combinations *Dt1/Dt2/TCP13*‐C and *Dt1/Dt2/TCP13*‐T, and the determinate growth habit combinations *dt1/Dt2/TCP*13‐C, *dt1/Dt2/TCP13*‐T, *dt1/dt2/TCP13‐*C, and *dt1/dt2/TCP13*‐T. Among them, *dt1/Dt2/TCP*13‐C and *dt1/Dt2/TCP13*‐T are rare alleles due to the low frequency of *dt1/Dt2*. Although it is more complex than that in RILs, the *Dt1/dt2/TCP13*‐T haplotype showed the highest plant height across all environments, while *Dt1/Dt2/TCP13*‐C haplotype exhibited the lowest plant height in these soybean accessions. Moreover, *TCP13*‐T contributed to plant height only under indeterminate and semi‐determinate growth habit condition, while under the determinate growth habit, no significant differences in plant height were found between the *TCP13*‐T and *TCP13*‐C genotypes (Figure 9).

Since plant height is related not only to plant architecture but also to yield, a yield‐related trait, pod number, was also incorporated into the haplotype analysis of the RILs. While the trends were not fully consistent with those for plant height, plants carrying haplotypes associated with increased height generally produced more pods than those with dwarf‐associated haplotypes (Figure S2). Notably, the best‐performing haplotype combination (*Dt1*/*dt2*/*TCP13‐T*) formed significantly more pods than the poorest‐performing combination (*dt1*/*Dt2*/*TCP13‐C*) consistently across all environments (Figure S2). These findings suggest that plant height may exert a significant effect on yield.

## 4 DISCUSSION

An ideal plant architecture improves photosynthetic efficiency, increases planting density, and enhances crop yield (Morinaka et al., 2006). In soybean, plant height not only affects architecture but is also a critical agronomic trait influencing yield (L. Chen et al., 2021; Zhai et al., 2022). To investigate this trait, we evaluated an RIL population across multiple environments. Plant height exhibited significant variation with a continuous distribution (Table 1; Figure 1). A total of 13 QTL were identified for plant height, most of which were environment‐specific (Figure 2), suggesting strong genotype‐by‐environment interactions. SoyBase database revealed that all loci overlapped with previously reported QTL (Q. Chen et al., 2007; I. S. Kim et al., 2021; K. S. Kim et al., 2012; Lark et al., 1995; Lee, Bailey, Mian, Carter, et al., 1996; Lee, Bailey, Mian, Shipe, et al., 1996; W. Li et al., 2008; Mansur et al., 1993, 1996; Orf et al., 1999; Palomeque et al., 2009; Pathan et al., 2013; Reinprecht et al., 2006; Specht et al., 2001; D. Sun et al., 2006; D. Wang et al., 2004; W. K. Zhang et al., 2004). This suggests that these genomic regions have significant effects on soybean plant height and need to be studied in‐depth, which enhances the reliability of our results.

We summarized colocalized or closely positioned QTL on the same chromosome and identified four stable QTL associated with soybean plant height across multiple environments (Table 2). These QTL showed consistency across multiple environments, indicating their stability and application potential in breeding programs. The genomic regions of three of the four stable QTL (*qPH6‐1*, *qPH18*, and *qPH19‐2*), which explained high phenotypic variances, were further annotated and analyzed between two parental lines, and three strong candidate genes underlying the three major stable loci were suggested based on SNPs, expression patterns, and functional annotation. For *qPH6‐1* locus, *Glyma.06G204300* (encoding *TCP13*, a TCP transcription factor) emerged as a strong candidate. The TCP family governs diverse developmental processes (Feng et al., 2018), with functional specialization across subfamilies: Class I TCPs modulate plant height through cell division regulation in Arabidopsis (Davière et al., 2014); CIN members control leaf morphogenesis (Urano et al., 2022); and CYC/TB1 subfamily proteins influence floral organogenesis and branching (J. Wang et al., 2010). Furthermore, *TCP13*, also named *QNE1*, was reported to be a key flowering regulator determining the length of the vegetative period in soybean cultivars (Xia et al., 2022). The C > T SNP at position 1063 in the coding region of this gene is the main contributor to the variation in flowering time and pod maturity date (Xia et al., 2022). In our study, we also detected the C > T SNP at position 1063 between two parents (Table 3; Figure 3). Therefore, we speculated that *TCP13*, a regulator for flowering time in soybean, may contribute to plant height by regulating the length of vegetable period. The effect of *TCP13* on plant height was further confirmed in the association study, where the RILs and soybean accessions harboring *TCP13‐T* (at position 1063 in the coding region of *TCP13*) showed higher plant height than those harboring *TCP13*‐C (Figures 4C and 5C). Within the *qPH18* interval, we identified *Glyma.18G273600* (*Dt2*) as a strong candidate gene, which encodes a MADS‐box transcription factor. Previous studies have established that *Dt2* regulates semi‐determinate growth habit in soybean, with pleiotropic effects on branch number and flowering time (Kou et al., 2021; Liang et al., 2022). SNP analysis revealed three vital variations in this gene between parents. The SNPs in promoter region have been reported to be significantly associated with the main function of *Dt2* (Liang et al., 2022). Given the known functional importance of *Dt2* in growth habit and flowering, we propose that *Dt2* serves as a strong candidate gene of *qPH18* locus for plant height. Subsequently, we confirmed the effect on *Dt2* on plant height through an association study in the RILs and soybean accessions, where *dt2* accessions exhibited higher plant height than *Dt2* (Figures 4B and 5B). The candidate gene *Glyma.19G194300* (*Dt1*) within the genomic region of *qPH19‐2* encodes a TFL1 protein, a key regulator of indeterminate growth in soybean (Z. Tian et al., 2010). When the *Dt1* gene is present, plants exhibit an indeterminate growth habit, meaning that the main stem continues to grow after flowering (X. Li et al., 2024; Z. Tian et al., 2010). In the current study, a nonsynonymous SNP of *Glyma.19G194300* between parental lines (ZH35 and ZH13) leads to an allelic change from *Dt*1 to *dt1*, resulting in the growth habit change from indeterminate to determinate. Since growth habit has pronounced effects on plant height (Clark et al., 2023). *Dt1* is assumed as strong candidate gene for *qPH19‐2*. Association analysis in the RILs and soybean accessions confirmed *Dt1* has significant influence on plant height, where the plant height of *Dt1* accessions was higher than that of *dt1* (Figures 4A and 5A).

The pyramiding effect of multiple genes governing a specific trait provides more comprehensive understanding for the complex connection of these genes (J. Zhang et al., 2024). In addition, the pyramiding effect could be applied in breeding programs through marker‐assisted selection (Sivabharathi et al., 2024). Soybean stem growth habit, which contributes to plant height, is primarily regulated by the *Dt1* and *Dt2* through control of apical dominance transition (Bernard, 1972). The *Dt1* controls the indeterminate growth habit. Plants harboring *Dt1* exhibit indeterminate growth. *Dt2* encodes a MADS‐box transcription factor that directly binds to the promoter of the *Dt1* to repress its expression. Therefore, plants bearing *Dt1/Dt2* exhibit a semi‐determinate growth habit (Xiong et al., 2023). Homozygous plants with the recessive *dt2* allele exhibit an indeterminate growth habit; the plants bearing *Dt1/dt2* display indeterminate growth. Moreover, in the genetic background of *dt1/dt1*, the effect of *Dt2/dt2* is masked, and all plants exhibit a determinate growth habit (Ping et al., 2014; D. Zhang et al., 2019). In this study, the pyramiding effect of *Dt1* and *Dt2* was performed in both the RIL population and 1484 soybean accessions. The plant heights of the four haplotype combinations were significantly different from each other in RILs. A decreasing trend for plant height was observed, with genotype‐specific height rankings ordered as *Dt1*/*dt2* > *Dt1*/*Dt2* > *dt1*/*dt2* > *dt1*/*Dt2* (Figure 6), which is consistent with the growth habits from indeterminate to determinate. Surprisingly, significant differences were observed between *dt1/dt2* and *dt1/Dt2* across four environments, suggesting *dt2* has positive effect on plant height even in the *dt1* background. In 1484 soybean accessions, the plant heights of accessions harboring *Dt1*/*dt2* were also significantly higher than those harboring *Dt1*/*Dt2*. However, no significant differences were observed in plant height between *dt1*/*dt2* and *dt1*/*Dt2*, although *dt1*/*dt2* exhibited slightly higher plant height than *dt1*/*Dt2* (Figure 8). This result may be attributed to synergistic selection of these two genes in the domestication and improvement process, with the combination of specific genotypes likely being selected, resulting in an extremely low frequency of *dt1*/*Dt2* genotype in soybean accessions (0.34%‐0.54% across environments).

The flowering time also affects plant height (Cai et al., 2020; Qin et al., 2023). *TCP13* (*QNE1*) interacts with soybean florigen *GmFT2a* and *GmFT5a* to promote flowering (Xia et al., 2022). Furthermore, different combinations of *TCP13* and *E4* (another flowering gene) alleles may result in a major influence on plant height (Xia et al., 2022). When the *TCP13* allele was subjected to pyramiding analysis with *Dt1* and *Dt2*, we found *TCP13*‐T positively contributes to plant height under each combination of *Dt1* and *Dt2* alleles in RILs (Figure 7), suggesting an additive effect of this allele on plant height. In the 1484 soybean accessions, however, the positive effect of *TCP13*‐T on plant height was only observed under indeterminate and semi‐determinate growth habit genotypes in soybean accessions (Figure 9), suggesting a potential epistatic interaction between *dt1* and *TCP13* for plant height regulation during domestication and improvement process.

We also evaluated the effect of combining three strong candidate genes for plant height on a yield‐related trait. In the RIL population, pod number varied significantly among lines with different haplotype combinations of *Dt1*, *Dt2*, and *TCP13*. RILs carrying the haplotype combination associated with the shortest plant height tend to produce fewer pods than those with combinations linked to greater plant height, suggesting that these haplotype combinations may also influence soybean yield (Figure S2).

Together, by integrating QTL mapping and haplotype analysis, we identified favorable plant‐height alleles from both parents, which exhibited additive effects. The tall parent, ZH35, carried the *Dt1* allele associated with increased plant height, consistent with its overall tall phenotype, yet it also possessed the height‐reducing alleles *Dt2* and *TCP13‐*C. Conversely, the shorter parent ZH13, carried the height‐reducing *dt1* allele, but it also harbored the height‐increasing alleles *dt2* and *TCP13*‐T. Thus, rather than relying on single‐gene effects, the combined action of favorable alleles at *Dt1*, *Dt2*, and *TCP13* more effectively enhanced stem growth habit and plant height variation. This makes marker‐assisted selection of these loci a feasible strategy for optimizing plant architecture in soybean breeding programs.

## 5 CONCLUSION

In this study, 13 QTL were identified associated with soybean plant height, among which four QTL (*qPH‐5*, *qPH6‐1*, *qPH18*, and *qPH19‐2*) were consistently detected across environments. We performed candidate gene mining for three stable QTL with high contribution to plant height. Based on SNP annotation within the candidate intervals, expression profiles, and functional predictions of genes, *Dt1*, *Dt2*, and *TCP13* were proposed as key candidate genes regulating plant height. Individual and combined haplotype analysis of these genes demonstrated that variations in each gene significantly influenced plant height. Moreover, different combinations of haplotypes among the genes had distinct effects on plant height, highlighting their potential for breeding soybean varieties with ideal plant architecture. Overall, this study provides valuable insights and specific targets for plant height improvement in soybean breeding practice.

## AUTHOR CONTRIBUTIONS

**Dan Sha**: Data curation; investigation; methodology; software; validation; writing—original draft. **Zhenzhen Zhang**: Data curation; investigation; methodology; validation. **Yongzhe Gu**: Data curation; investigation; resources. **Shengrui Zhang**: Data curation; investigation. **Aimal Nawaz Khattak**: Investigation; writing—review and editing. **Yitian Liu**: Data curation; investigation. **Caiyou Ma**: Investigation. **Meng Hu**: Data curation; investigation. **Jimeng Niu**: Data curation; investigation. **Linfeng Yu**: Investigation. **Shibi Zhang**: Data curation; investigation; resources. **Azhar Iqbal**: Investigation. **Ahsan Muhammad**: Investigation. **Jing Li**: Data curation; investigation. **Junming Sun**: Conceptualization; funding acquisition; project administration; writing—review and editing. **Rongxia Guan**: Conceptualization; writing—review and editing. **Bin Li**: Conceptualization; funding acquisition; project administration; supervision; writing—review and editing.

## CONFLICT OF INTEREST STATEMENT

The authors declare no conflicts interests.

## Supporting information




**Figure S1** The relative expression levels of three strong candidate genes in shoot apex between parents Zhonghuang35 (ZH35) and Zhonghuang13 (ZH13), A) *Dt1*, B) *Dt2*, C) *TCP13*.** indicates significance at *P* < 0.01 level, *** indicates significance at *P* < 0.001 level.
**Figure S2** Pyramiding effect of *Dt1*, *Dt2*, and *TCP13* on pod number in the RIL population. The bars show the pod numbers of major haplotypes (more than five accessions) with standard errors (SE). Different lowercase letters above bars denote statistically significant differences at *P* < 0.05.

**Table S1** The primers used in qPCR analysis for *Dt1*, *Dt2*, and *TCP13*.
**Table S2** All QTL for soybean plant height across five environments.
**Table S3** List of genes within the genomic regions of plant‐height associated QTL, *qPH6‐1*, *qPH18* and *qPH19‐2*.
**Table S4** Missense and upstream SNPs within the genomic regions of plant‐height associated QTL, *qPH6‐1*, *qPH18*, and *qPH19‐2*.
**Table S5** The expression profiles of candidate genes for plant height within *qPH6‐1*, *qPH18* and *qPH19‐2* intervals.

## Data Availability

Additional supporting information can be found online in the Supporting Information. The genotypic and phenotypic data for haplotype analysis of *Dt1*, *Dt2*, and *TCP13* in 1484 soybean accessions were downloaded from the SoyOmics database (https://ngdc.cncb.ac.cn/soyomics/haplotype/).
