# Supplementary material for: High‐resolution quantitative trait loci mapping and pyramiding effects of candidate genes for plant height in soybean
Source: Plant Genome. 2026 Feb 24;19(1):e70207. doi: 10.1002/tpg2.70207 (PMC12930336; doi:10.1002/tpg2.70207)
Supplement: Supplementary file 1 — Figure S1 The relative expression levels of three strong candidate genes in shoot apex between parents Zhonghuang35 (ZH35) and Zhonghuang13 (ZH13), A) Dt1, B) Dt2, C) TCP13.** indicates significance at P < 0.01 level, *** indicates significance at P < 0.001 level. Figure S2 Pyramiding effect of Dt1, Dt2, and TCP13 on pod number in the RIL population. The bars show the pod numbers of major haplotypes (more than five accessions) with standard errors (SE). Different lowercase letters above bars denote statistically significant differences at P < 0.05. [file TPG2-19-e70207-s002.docx]

**
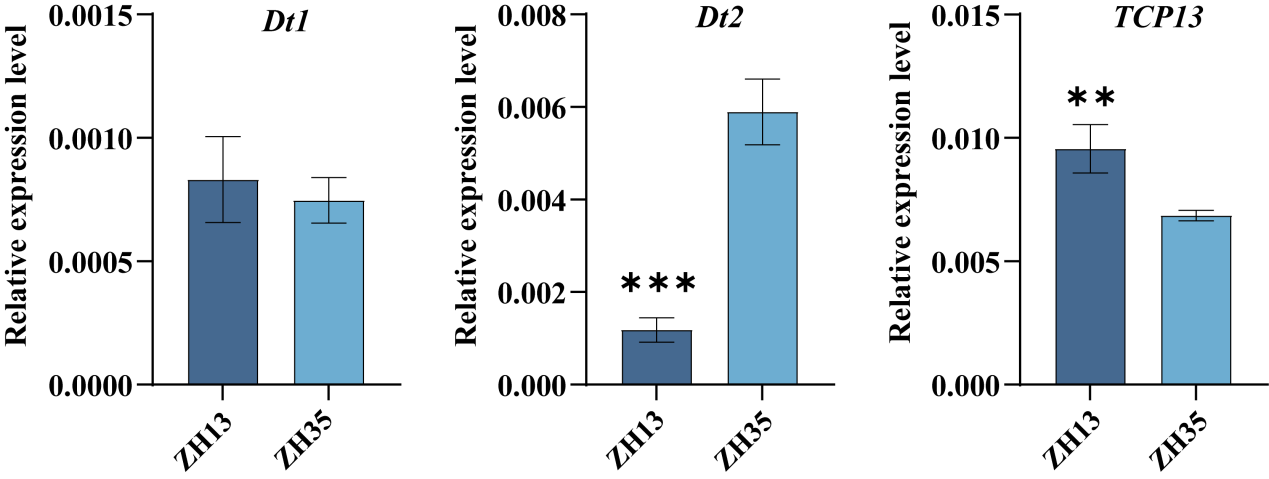
Fig. S1** The relative expression levels of three strong candidate genes in shoot apex between parents Zhonghuang35 (ZH35) and Zhonghuang13 (ZH13), A) *Dt1*, B) *Dt2*, C) *TCP13*.** indicates significance at *P* < 0.01 level, *** indicates significance at *P* < 0.001 level.

**
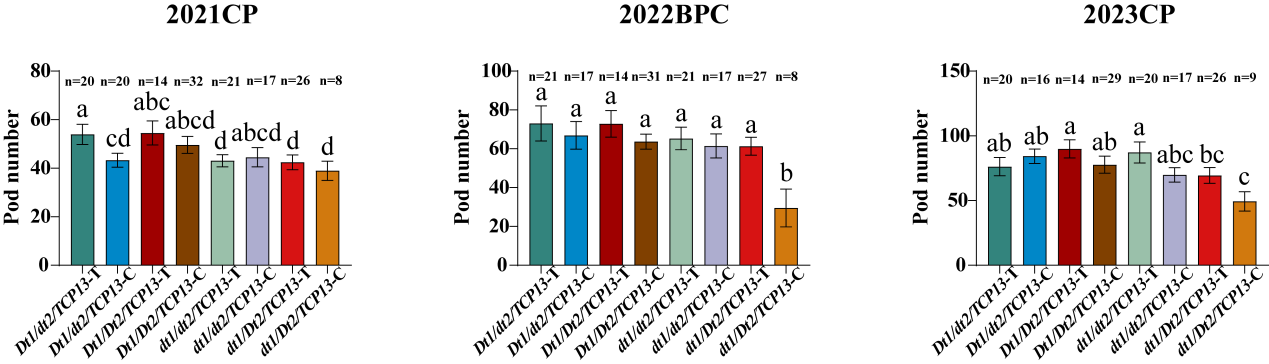
Fig. S2** Pyramiding effect of *Dt1*, *Dt2,* and *TCP13* on pod number in the RIL population. The bars show the pod numbers of major haplotypes (more than five accessions) with standard errors (SE). Different lowercase letters above bars denote statistically significant differences at *P* < 0.05.
